# Laying the foundation for *iCANmeditate*: A mixed methods study protocol for understanding patient and oncologist perspectives on meditation

**DOI:** 10.1371/journal.pone.0290988

**Published:** 2024-08-22

**Authors:** Yasmin Lalani, Alexandra Godinho, Kirsten Ellison, Krutika Joshi, Aisling Curtin Wach, Punam Rana, Pete Wegier

**Affiliations:** 1 Humber River Health Research Institute, Humber River Health, Toronto, Ontario, Canada; 2 Humber River Health, Toronto, Ontario, Canada; 3 Department of Family and Community Medicine, Toronto, Ontario, Canada; 4 Institute of Health Policy, Management and Evaluation, Toronto, Ontario, Canada; PLOS ONE, UNITED KINGDOM OF GREAT BRITAIN AND NORTHERN IRELAND

## Abstract

**Background:**

People with cancer experience heightened levels of stress and anxiety, including psychological or physical. In recent years, digitally delivered complimentary therapies, such as meditation, have gained attention in cancer research and advocacy communities for improving quality of life. However, most digital meditation resources are commercially available and are not tailored to the unique needs of cancer patients (addressing fears of recurrence). As such, this study lays the foundation to co-design a publicly available digital meditation program called *iCANmeditate* that contains cancer-specific meditation content.

**Aims:**

To understand: (1) cancer patients’ perceptions and practices of meditation, as well as their needs in addressing the stress that accompanies their cancer diagnosis and (2) current knowledge of meditation and prescribing trends amongst oncologists in Canada.

**Methods and analysis:**

A mixed-methods design comprised of online patient and oncologist surveys and interviews with patients will be used. Survey data analysis will use multivariate logistic regressions to examine predictors of: (1) interest in using a meditation app among patients and (2) prescribing meditation among oncologists. Patient interviews will gather insights about the contexts of daily living where meditation would be most beneficial for people with cancer; this data will be analyzed thematically.

**Discussion:**

The results of this study will inform iterative co-design workshops with cancer patients to build the digital meditation program *iCANmeditate*; interview results will be used to develop vignettes or “personas” that will supply the initial stimulus material for the iterative co-design workshops. Once the program has been finalized in partnership with cancer patient participants, a usability and pilot study will follow to test the functionality and efficacy of the tool. Results from the oncologist survey will form the basis of knowledge mobilization efforts to facilitate clinical buy-in and awareness of the benefits of meditation to cancer patients.

## Introduction

Most people with cancer experience heightened levels of stress, which can often continue post-recovery for many patients [1–4]. This stress can take on several physiological and physical forms and may correlate with symptoms such as anxiety, depression, fatigue, pain and insomnia [5]. Cancer patients have become increasingly interested in using complementary therapies for reducing side effects, and improving overall well-being [6, 7].

One complementary therapy that has shown positive outcomes for patients to manage their cancer-related symptoms is meditation [8–11]. Meditation is a mind-body practice that has gained widespread appeal in healthcare and clinical practice guidelines as an evidence-based therapy for improving mental health (i.e. anxiety, depression, physiological distress), physical health (i.e. sleep, fatigue, pain, feeling sick), and overall quality of life in those affected by cancer [12, 13].

The uptake of meditation in cancer populations is also rising across different types and stages of cancer marked by surveys demonstrating over one third of patients use it as a form of complementary therapy, [6] and via the growing number of studies examining its efficacy across different types and stages of cancer [14–18]. Moreover, a general shift towards offering meditation as a digital resource has been observed within the last decade, as compared to traditional in-person sessions. Online or smartphone-based meditation apps offer cancer patients greater flexibility and access to care, by removing some of the reported challenges of attending face-to-face classes such as: lack of nearby services, competing interests for time (e.g., appointments, caregiver availability, childcare), and committing to travel and lengthy sessions when distressed and fatigued [19–21]. In addition, recent findings have also shown a preference among cancer patients for using smartphone applications to learn about support care services, particularly among younger and non-white patients thereby making digital meditation a potentially viable support service [22].

Commercially available digital meditation tools have shown promise in cancer patients, with initial efficacy trials demonstrating these tools to be comparable to in-person sessions in terms of acceptability, feasibility, and positive outcomes for cancer populations [23–25]. However, despite the numerous publicly available digital meditation tools on the market (e.g., Calm^TM^, Headspace^TM^*)* that have shown promise in cancer patients, few are tailored to the specific needs and preferences of people affected by cancer [15, 21, 26]. Indeed, findings from a recent study suggest that cancer patients would prefer to use digital meditation tools that are optimized to their unique needs and preferences [8, 27]. In particular, Huberty and colleagues revealed that cancer patients who used the Calm^TM^ app for meditation, have expressed modifications to Calm^TM^ to contain more specific information about how to center on positive feelings, how to manage negative feelings associated with their cancer journey, and content that promotes movement-based activities [27].

To address this gap and provide a publicly available solution, we propose to co-design a digital meditation tool called *iCANmeditate* that is tailored to the specific needs of cancer patients and leverages an existing hospital-based digital platform. We will be conducting this study with the guidance of a medical oncologist and certified meditation teacher. To accomplish this, we must first understand: (1) patient perceptions and practices of meditation as well as the needs and concerns of cancer patients’ experiences with stress and coping with their diagnosis and (2) current knowledge of meditation and prescribing trends amongst oncologists. To date, only one study has examined the knowledge and perceptions of meditation among cancer patients in preparation for designing a tool [28]. Overall, this study found that while the benefits of meditation were generally understood by cancer patients, one of the main barriers to engaging with meditation was a lack of perceived knowledge. In addition, these scholars found that experience with meditation use seemed to vary by gender, age, and education level; however, interest in a digital resource was highly correlated with reporting a higher level of stress [28]. Although these findings are important for understanding the characteristics of cancer patients who may be more likely to use and benefit from a digital meditation tool, this study was limited to melanoma patients and is not generalizable to all cancer subtypes.

## Aim of the study

We propose to conduct a survey and semi-structured interviews on patient knowledge, prior use, and attitudes towards meditation as it relates to their experiences of stress and coping with their diagnosis. In addition, we also propose to survey Canadian oncologists on their current knowledge and attitudes towards meditation, as no study to date has been conducted in this area to our knowledge. The survey will ask oncologists about their awareness of different digital meditation resources and clinical practice guidelines, as well as current practices in prescribing meditation to their patients. As this study is exploratory in nature, no a priori hypotheses were made.

## Methods

### Participants

#### Patients

Outpatients attending the Humber River Health Cancer Clinic will be invited to take part in a survey about well-being, including strategies for coping with their stress and anxiety. Eligibility criteria will consist of being 18 years of age or older, currently receiving treatment for cancer and English-speaking; for interview participants they will have indicated on the survey that they would be interested to engage in focus groups; however, interviews will be conducted instead due to scheduling constraints. Individuals will not be excluded based on type or stage of cancer, however those with cognitive impairments, psychiatric illness, or are too distressed to participate as identified by clinicians will be excluded. The following types of cancer will be excluded from the sample as they are not currently treated at the clinic: head, neck, sarcoma, skin, melanoma, gynecological cancers.

#### Oncologists

Members of the Canadian Association of Medical Oncologists (CAMO) will be invited to take part in a brief online survey about their perspectives on prescribing meditation to improve quality of life in cancer patients. CAMO is comprised of over 350 members that include: medical oncologists, residents, fellow, and associates. In addition, email invitations for the survey will also be sent to oncologists via professional contacts of the authors at various hospitals / academic institutions and to any oncologists whose contact information is publicly available. Eligibility criteria will consist of being 18 years of age or older, having completed their fellowship, and practicing medical oncology.

### Study design and procedures

#### Patient survey and interviews

A cross-sectional survey will be administered, targeting eligible participants identified through Humber River Health’s Cancer Clinic outpatient treatment appointment lists; clinical eligibility will be determined in consultation with a clinical nurse coordinator. Recruitment will take place in the cancer clinic treatment area, with research staff systematically approaching all patients in treatment bays who have not been identified by nursing staff as being on airborne precautions, significantly distressed, or cognitively unable to provide consent. Patients will be invited to participate in a survey about cancer patient’s well-being, including experiences of stress, anxiety, and quality of life. To ensure that patients in the clinic understand research activities are not a part of standard clinical care, posters will be placed in the cancer clinic waiting room. To reduce self-selection biases (i.e., recruiting only participants who are interested in meditation), and capture a wide range of patients’ perceptions and barriers towards meditation, participants will be blinded to the purpose of the survey. Consent forms and recruitment scripts will not include any mention of meditation. Interested potential participants will be directed to an online consent form by research staff on their individual integrated bedside terminal (i.e., tablet); this is a confidential and private tablet available to each patient receiving treatment in the cancer clinic. Informed consent will include an optional consent to be contacted for future studies and anonymous data sharing (i.e., open science). To ensure consent is informed, potential participants will be provided with the opportunity to ask questions and express concerns to research staff, with teach-back strategies being used as needed [29]. Those who complete the consent form will be redirected to an online survey. Upon survey completion participants will be debriefed about the true aims of the study (i.e., to understand patient perspectives and experience with meditation in preparation of developing a digital meditation tool) and will be provided with a patient resource about meditation. If participants feel some discomfort in reflecting about their wellbeing and quality of life even after they complete the survey, there is a distress centre phone number indicated on the consent form. In addition, all participants who complete the survey will be provided with a $5 physical gift card as a token of appreciation.

Participants who indicate on the survey that they would be interested in using a digital meditation resource and participating in online focus groups to help develop a cancer-specific meditation program will be considered for participation in virtual interviews. Participant characteristics and experience with meditation will be used to guide the selection of participants; a maximum of 15 participants will be recruited for individual interviews. Participants who are selected for participation will be emailed a copy of the consent form outlining the study in greater detail. To ensure consent is informed, consent forms will be reviewed with participants prior to the scheduled interview, and verbal consent will be obtained. All participants who engage in interviews will be compensated with a $35 electronic gift card. All interviews will be video, and audio recorded as well as transcribed verbatim. The video files will be permanently deleted, and the audio files will be kept for analysis. Risks of participating in interviews may be momentary discomfort of talking about their cancer experiences; if participants still feel distress after the interview, they may refer to the Distress Centres of Greater Toronto phone number indicated on the consent form as a way to seek professional help. All identifying information from audio transcripts will be removed.

#### Oncologist survey

Medical oncologists who are members of CAMO will be invited to complete a survey about their perspectives and knowledge of meditation for improving quality of life in cancer patients via an email survey blast. To promote recruitment efforts, among CAMO members who are sent the survey via an email blast up to two reminders will be sent 1-week post-initial survey email blast, and the first reminder, respectively. Those who are interested in completing the survey will be directed to an electronic consent form via a link contained in the invitation email. Potential participants who complete the electronic consent will be automatically directed to the survey on their browser. Upon completion of the survey, oncologists will be provided with a meditation resource that they can view and download. Participants will be compensated with a $5 electronic gift card.

Ethics approval for the research methods used in this study, including the oncologist survey, patient survey/interviews was provided by Veritas Institutional Review Board.

### Meditation resources

All meditation resources in this study provide a plain-language overview of meditation and draw on existing meditation resources provided by the Canadian Cancer Society, the National Center for Complementary and Integrative Health, and the Memorial Sloan-Kettering Cancer Centre. All materials have been reviewed for accuracy by the clinical subject matter lead for meditation.

#### Patient meditation resource

Following the completion of the patient survey, participants will be offered a tri-fold brochure that provides a plain-language overview of what meditation is and how it has been shown to benefit cancer patients. The brochure will also provide them with a link and QR code to the oncologist/meditation teacher’s website, where they will have access to additional resources and information.

#### Oncologist meditation resource

A one-page document will also be made available at the end of the survey to oncologists who express interest in learning more about meditation (i.e., respond “*yes”* to survey item asking about interest in meditation resources). This electronic pdf will provide oncologists with a plain-language overview of what meditation is and how it has been shown to benefit cancer patients. The document will also provide them with references to current clinical guidelines and a list of relevant online resources and recommended patient resources.

### Measures and qualitative methods

#### Patient survey measures

The survey will assess a variety of demographic characteristics that have been associated with meditation practice such as: age, sex at birth, education level, employment, and religion. In addition, clinical characteristics such as cancer diagnosis details (i.e., type, stage, date of diagnosis) and mental health outcomes will also be assessed [30, 31].

*Mindfulness*. Everyday mindfulness will be measured using the Cognitive and Affective Mindfulness Scale-Revised (CAMS-R), which measures the following aspects of mindfulness: present-focused attention, awareness, and nonjudgmental acceptance of thoughts and emotions [32]. This tool has been shown to be suitable for use in meditating as well as non-meditating samples, and is commonly used in cancer patient samples to assess individual differences in dispositional mindfulness [28]. This study will use the brief 10-item version of the tool, where scores range from 10 to 40,with higher scores representing greater self-reported mindfulness.

*Experience With Meditation*. A series of questions have been developed by the team, in consultation with the oncologist and meditation expert, to assess patients’ practice of meditation. In particular, participants will be asked about: type of meditation practiced (e.g., mindful, transcendental); resources used (e.g., apps, books, websites); tools to assist in meditating (e.g., incense, mat, bells); duration and frequency of practice (months/years, daily/weekly/monthly); and changes in meditation since their cancer diagnosis (decision to meditate/frequency). In addition, participants will also be asked about current interest in learning more about meditation, continuing their current practice, and the extent to which they feel their practice of meditation is helpful.

*Barriers to Meditation*. Perceived barriers for meditating among cancer patients who currently meditate, versus those who do not practice meditation will be assessed using the Determinants of Meditation Practice Inventory–Revised (DMPI-R) [33].This briefer version of the full DMPI, [34] is a psychometrically validated 12-item self-report tool that measures 4 subscales of barriers to meditating: low perceived benefit, perceived inadequate knowledge, perceived pragmatic barriers, and perceived socio-cultural conflict. Each subscale is comprised of 2–4 items that are scored on a 5-point Likert-type scale, with higher scores indicating a higher perception of that perceived group of barriers.

*Knowledge of Meditation*. Patient knowledge of meditation will be assessed using a series of questions that gauge general perceptions of meditation. A set of 10 items have been developed by Russell et al. [28] to assess knowledge of meditation. Survey respondents are asked to rate their agreement with 10 statements (i.e., seven facts and three misconceptions that are reverse scored), using the response options 1 “Agree”, 0 “Don’t know” or -1 “Disagree. Overall, higher total scores indicate greater knowledge of meditation.

#### Individual interviews

The purpose of the interviews will be to explore participants’ (1) experiences, perceptions, and preferences regarding meditation and (2) how and under what contexts their practice of meditation has (or has not) helped the cope with their cancer diagnosis. The interview guide will ask general questions, followed by more specific key questions directly related to the research topic so that participants are given ample time to “warm up” and feel comfortable with the interviewer [35, 36]. Please see S1 Appendix for the interview guide.The interviewer will be a member of the research team with qualitative research expertise. Each interview will last between 30 and 90 minutes; however if a patient has more they want to share, then a second interview may be scheduled.

#### Oncologist survey measures

The survey will assess demographic characteristics such as participant age and sex as well as professional practice characteristics including main place of work, years and province of practice, and area of oncology specialty. To determine current practices around recommending alterative and complementary therapies for alleviating stress and anxiety among cancer patients, participants will be asked to identify any therapies they currently recommend from a pre-specified list of the most common forms (e.g., music therapy, meditation, acupuncture); an open-ended user option will also be included to capture therapy forms not listed.

*Meditation Recommendation Practices*. To assess the proportion of survey completers who specifically recommend meditation, an additional yes/no question will be asked to those who do not select meditation from the aforementioned list (i.e., “Have you *ever* recommended meditation to your patients?”). In addition, for those who respond that they do or have recommended meditation, further questions will assess the forms of meditation that have been recommended (i.e., types of meditation vs. delivery format), and how the recommendation was made. In order to better understand how oncologists recommend meditation to their patients, an item was developed to assess the physicians’ level of engagement in providing information and resources about meditation. This item will ask “Which statement best describes how you recommend meditation to your patients?” and provide the following response options: (a) I suggest meditating to help with stress and anxiety, (b) I recommend meditation to my patients and provide them with resources to help them learn and understand how to meditate, and (c) I explain the benefits of meditation to my patients and provide them with resources to help them learn and understand how to meditate.

*Knowledge of Meditation*. The survey will also aim to understand oncologists’ attitudes and knowledge of meditation, by asking the same series of questions outlined for patient participants; see above.

*Awareness of Practice Guidelines Endorsing Meditation*. The last component of the survey aims to understand oncologists’ general awareness for guidelines that currently recommend meditation to cancer patients. In 2018, the American Society for Clinical Oncology (ASCO), endorsed guidelines put forth by the Society for Integrative Oncology (SIO), recommending music therapy, meditation, stress management, and yoga for anxiety and stress reduction among breast cancer patients. Survey respondents will be presented with a partial screenshot of the practice guidelines and items will be asked to assess (1) knowledge/awareness for the guidelines, (2) if their recommendations of integrative therapies changed upon release of the guidelines, and if so what therapies they started recommending to their patients, and (3) for those without awareness for the guidelines, if becoming aware of them via the survey would influence their recommendation of integrative therapies moving forward as well as which specific therapies they will begin recommending to patients.

## Data management

The electronic platform, Qualtrics, used to collect consent and participant data for both the patient and oncologist online surveys ensures research participants’ privacy and confidentiality, and does not share data with third parties. Survey responses on Qualtrics are protected by Qualtrics’ Web Application Firewalls and their detection system that monitors for unauthorized users. Data will not be linked from other sources and will only be used for purposes outlined in the consent documentation. Patient and oncologist digital survey data will be stored in a secure computer file and will be deleted upon the completion of the study. A Master Linking Log that contains patients’ Study IDs and personal identifying information will be kept as a password protected file, accessible to the research team during the recruitment process only. Interview audio files will be sent to a transcription service using secure file transfer; transcripts will be anonymized and stored both in a locked office (if they are printed as paper copies) as well as digitally as password protected digital files. All study data (i.e., survey data /interview transcripts) will be stored securely for at least five years on Humber River Health’s encrypted cloud storage, after which the de-identified data will be deposited into Humber River Health’s Institutional Repository for a period of 10 years. The de-identified data will not be sold and will only be accessible to the study team.

## Data analysis and sample size calculations

### Patient survey data analysis and sample calculation

The primary analysis will be a prediction of who is interested in using a meditation app, applying multivariate Logistic Regression with two-sided p<0.05 as critical value. The covariates include age, anxiety level, perceived stress, and perceived mindfulness while the factors include sex and whether the patient uses alternate methods of stress management. In secondary analyses, we will use bivariate statistical analyses to identify relationships, interactions, or differences between the covariates/factors using T-tests and Spearman correlations. Based on the number of covariates/factors (k = 6), and a lower estimate of the proportion of the positive outcome variable (40%; v = 0.4), the minimum number of cases required is calculated by the formula (N = 10k/v), as determined by Peduzzi, et al. [37] This results in a calculated sample size of 150 for a meaningful logistic regression analysis.

### Patient interview data analysis

Thematic analysis of interviews will require a dynamic, non-linear, and iterative engagement with transcripts and selective listening of audio. All transcripts and audio files will be uploaded into NVivo 12 qualitative analysis software program as the majority of the analysis steps will be conducted in this program. To ensure that analysis is trustworthy, interview data will be analyzed by the qualitative lead in the research team. The approach to analysis will be iterative and conducted thematically. Data will be analyzed using thematic analysis until data saturation has been reached [38, 39].

The lead analyst will work with other members of the research team to discuss codes and themes that are under construction during the analysis process. The purpose of these discussions is twofold: (1) to achieve consensus regarding the meanings and definitions of codes as well as resulting themes to resolving and (2) to incorporate any non-verbal communicative data from the recording rather than solely the transcript. In doing so, we will eliminate as much as possible, the potential misleading interpretations of participants’ voices analyzed as words on the transcript only.

### Oncologist survey data analysis and sample calculation

The primary analysis will be a prediction of who prescribes meditation using multivariate Logistic Regression with two-sided *p*<0.05 as critical value. The covariates include age while the factors include sex and whether alternate therapies are recommended. In secondary analyses, we will use multiple regression to determine relationships between the covariates/factors. We will also test for interactions, particularly between whether alternate therapies are recommended and who prescribes meditation. Based on the number of covariates/factors (k = 3), and a lower estimate of the proportion of the positive outcome variable (20%; v = 0.2), the minimum number of cases required is calculated by the formula (N = 10k/v), where 10 is the minimum number of ‘events per variable’ as determined by Peduzzi, et al. [37] This results in a calculated sample size of 150 for a meaningful logistic regression analysis. As a result, all oncologists belonging to the Canadian Institution of Medical Oncologists (CAMO) will be given the survey.

## Discussion

### Significance of the proposed study

The study outlined in this protocol aligns with recent research in healthcare interventions and delivery that empowers patients to engage with digital solutions to manage their symptoms and enhance their wellbeing [40, 41]. Cancer patients in particular experience stress, pain, and decreased quality of life as a consequence of their diagnosis; some patients would therefore benefit greatly from a digital tool for symptom management. Thus, this study responds to the most recent call for online meditation programs for cancer to be tailor-made for cancer patients as opposed to standard meditation digital applications [8, 27].

This study protocol serves as the first step to address this gap; it outlines the rationale and procedures for understanding cancer patients’ perceptions and practices of meditation as well as their experiences of how meditation may have benefited them. In tandem with the quantitative and qualitative patient data, the survey results of practicing oncologists in Canada will aid in our understanding of their prescribing practices and state of meditation knowledge. Taken together, the patient and oncologist datasets will prepare the infrastructure needed for the next phase of the broader research goal—to co-design and pilot test an online meditation program called *iCANmeditate*.

The co-design process will be informed by vignettes generated from patient interview data. Vignettes are fictional representations or “personas” of individuals’ experiences of the topic under study and have practical applications in healthcare in co-design or participatory health research [42]. Vignettes will be presented to cancer patient participants during the development phase of this larger research program to: (1) develop content and a prototype of the online meditation tool on the hospital’s platform and (2) to test the usability of the meditation program. Co-design workshops will be run iteratively with feedback incorporated by participants until *iCANmeditate* is complete. A pilot study will follow the development of *iCANmeditate* to measure the tool’s efficacy.

### Strengths and limitations

One key strength of this study is our engagement with oncologists in Canada; there is a paucity of research that demonstrates the level of knowledge and prescribing behaviours of meditation as a complimentary integrative therapy from oncologists despite the fact that meditation is included in some clinical guidelines [12, 43]. Consequently, a long-term goal for the meditation program is to convey the value of meditation by involving oncologists so that meditation does not continue to be a “fringe” therapy with little clinical endorsement.

Possible limitations of this foundational study can be pinpointed. One limitation is that the patient population drawn from the hospital study site may not be representative of patients with cancer at a national or provincial level. For instance, the patient survey excludes participants whose command of English is weak and as such, survey results will only reveal data from an English-speaking group. Secondly, the hospital study site does not treat all types of cancer and thus, a comprehensive understanding of patients’ knowledge of meditation will exclude patients with head, neck, sarcoma, gynecological, skin and melanoma cancers. In a similar vein, the target population for the patient survey is patients who are actively in treatment and not patients who were recently diagnosed. Our rationale for excluding this group was to avoid adding an emotional burden to patients who may still be coming to terms with their cancer diagnosis. Finally, our patient sample will not include individuals who may identify as “survivors”; the concept of survivorship in cancer is an emerging field of study and consequently, outside the scope of this investigation of cancer patients currently in treatment.

In conclusion, this protocol outlines the research activities required to move to our next phase of research where continuous engagement with cancer patients during the co-design phase will produce a unique and meaningful meditation program that incorporates the specific needs of people with cancer.

## Supporting information

S1 Appendix (DOCX)
