# Supplementary material for: Laying the foundation for iCANmeditate: A mixed methods study protocol for understanding patient and oncologist perspectives on meditation
Source: PLoS One. 2024 Aug 22;19(8):e0290988. doi: 10.1371/journal.pone.0290988 (PMC11341032; doi:10.1371/journal.pone.0290988)
Supplement: S1 Appendix — (DOCX) [file pone.0290988.s001.docx]

Appendix A

| Topic Domain | Questions | Sample Prompts |
| --- | --- | --- |
| Introduction and purpose of interview (10 minutes) | - Welcome and explain the purpose of the interview | N/A |
| Opening Questions (10 minutes) | 1. What inspired you to participate in this interview? 2. Have you ever been involved in any cancer-related advocacy or support groups? If so, what was your experience like? |  |
| Key Questions on Meditation Experiences | 1. If you have experience with meditation, how did you first learn or “get into” meditation? 2. Think about a typical day. Can you describe a scenario or times of day or places when you have found meditation to help you or, if you have not meditated, what is a scenario or time of day that you think meditation might help calm you? 3. Describe your experiences with meditation either before and/or after your cancer diagnosis. 4. What benefits if any, have you experienced with practicing meditation? 5. Next, what barriers have you faced in practicing meditation? 6. If you have not meditated before, what prompted you to want to learn more? | - Are there specific types of meditation practices that you prefer? - How does meditation help you? What types of meditation do you normally practice and why? - Do these benefits come up when you are in specific places (e.g. waiting for appointments in the Cancer Clinic?) or when you are about to meet/have met with specific people? - Can you speak about meditation and any possible benefits to symptoms you experience? |
| Key Questions on Stresses and Cancer Diagnosis (30 minutes) | 1. What emotions you have been feeling the most since your diagnosis? 2. What kinds of challenges have you been facing in your everyday life as a result of your cancer diagnosis? 3. In what ways (if any) has meditation helped you live with cancer? 4. Do any of you use other complimentary therapies to improve your quality of life? If so, how does meditation compare to these other therapies? | - How would meditation help in coping with these emotions if at all? - If meditation has helped you, describe how it has helped with your stress or anxiety or physical symptoms. - If meditation has not helped you at all, please describe your reasons. |
